# Comprehensive Identification of miRNAs and circRNAs in the Regulation of Intramuscular and Subcutaneous Fat Deposition in Meat Ducks

**DOI:** 10.3390/genes16101208

**Published:** 2025-10-14

**Authors:** Zhixiu Wang, Tingting Zhou, Wenshuang Liang, Qianqian Song, Yong Jiang, Hao Bai, Guohong Chen, Guobin Chang

**Affiliations:** 1 Key Laboratory of Animal Genetics and Breeding and Molecular Design of Jiangsu Province, Yangzhou University, Yangzhou 225009, China; 2 Management Office of the South Campus, Taishan University, Taian 271000, China

**Keywords:** meat duck, subcutaneous fat, intramuscular fat, circRNAs, miRNAs, ceRNA

## Abstract

Fat deposition is an important factor that affects meat production and its quality in livestock animals, including poultry. Non−coding RNAs (ncRNAs) play an important role in duck fat deposition. This study aims to systematically identify key regulatory molecules involved in fat deposition in 8−day−old Cherry Valley ducks through transcriptomic sequencing across four sample groups: intramuscular pre−adipocytes (IMP−0), intramuscular adipocytes after 4 days of induction (IMP−4), subcutaneous pre−adipocytes (SCP−0), and subcutaneous adipocytes after 4 days of induction (SCP−4). Differential expression analysis preliminarily identified several circRNAs and miRNAs differentially expressed during adipocyte differentiation, including novel_circ_000012, novel_circ_000037, novel_circ_000089, as well as miR−501−y, miR−378−y, and miR−3968−y. Further co−expression network analysis revealed that the network constructed during intramuscular adipocyte differentiation comprised 17 nodes and 39 edges, while the network constructed during subcutaneous adipocyte differentiation was larger, containing 39 nodes and 50 edges. Based on connectivity screening, we identified several key miRNAs, such as novel−m0630−5p, novel−m0485−5p, novel−m0672−5p, miR−5126−y, and miR−1408−y. Notably, this study uncovered several novel ceRNA regulatory axes during intramuscular and subcutaneous adipocyte differentiation, including novel_circ_001327/miR−141−y/*Zdhhc1*, novel_circ_002268/miR−2478−y/*ACLY*, and novel_circ_002268/miR−3963−x/*ACLY*. These findings provide crucial molecular insights into the specific deposition mechanisms of intramuscular versus subcutaneous fat in meat ducks, offering valuable targets for molecular breeding programs aimed at improving meat quality.

## 1. Introduction

Duck meat, as a significant poultry product, has become a globally favored “health food” due to its high protein, low fat, and low cholesterol characteristics [1]. China, as the world’s largest duck meat producer, holds a particularly prominent position in this industry. With consumption upgrades, the market demands higher standards for meat duck quality, where fat composition and distribution are core factors determining meat nutritional value, flavor, and Palatability.

Among the numerous factors determining meat quality, the distribution of fat deposition is crucial, with intramuscular fat and subcutaneous fat playing distinctly different roles [2]. Intramuscular fat forms marbling patterns and is a key factor determining meat tenderness, juiciness, and flavor [2,3]. However, in commercial duck breeding, intramuscular fat deposition is often accompanied by excessive accumulation of subcutaneous fat. The latter not only reduces feed conversion efficiency [4,5] but also compromises carcass quality and economic value [6]. Therefore, how to specifically enhance intramuscular fat while suppressing subcutaneous fat deposition has become a core challenge in current meat duck breeding.

Fat deposition in livestock and poultry is a complex and sophisticated programmed biological process that is influenced by multiple factors, including a series of transcription factors, signaling pathways, and network regulation [7]. In recent years, non-coding RNAs (ncRNAs) have been demonstrated to play a central role in this process. Among them, microRNAs (miRNAs) serve as key post-transcriptional regulators, and their crucial roles in lipid metabolism and adipocyte differentiation in livestock and poultry have been extensively reported [8,9,10,11]. Concurrently, circular RNAs (circRNAs), another functionally rich class of ncRNAs, exert significant regulatory effects on the dynamic equilibrium of fat deposition and lipid metabolism [12,13]. This occurs through the classic mechanism of acting as “miRNA sponges,” competitively binding miRNAs and thereby releasing their inhibitory effects on downstream target genes [14,15]. Notably, in modern livestock production, the ideal deposition of intramuscular fat is often accompanied by excessive accumulation of subcutaneous fat [16,17], a contradictory phenomenon that directly impacts dressing percentage and economic efficiency. However, the biological mechanisms driving the differential deposition of these two fat tissues, particularly the ceRNA−based regulatory network, remain poorly understood.

Therefore, this study aims to use meat ducks as a model. Through transcriptome sequencing technology, we will screen for key differentially expressed miRNAs and circRNAs in subcutaneous fat versus intramuscular fat and construct their ceRNA regulatory networks. This research endeavors to reveal the molecular mechanisms underlying subcutaneous fat-specific deposition, providing a theoretical basis for achieving the precise breeding goal of “high intramuscular fat, low subcutaneous fat” in future breeding work, thereby advancing the development of high-quality meat duck breeds.

## 2. Materials and Methods

### 2.1. Animals and Samples Preparation

Cherry Valley ducks used in this study were purchased from Shuyang Zhongke Seed Poultry Co., Ltd. (Suqian, China). Ten 8−day−old Cherry Valley ducks were randomly selected, with no gender specification, and were in good health. For the first 8 days after hatching, the ducks had free access to feed and water. Subcutaneous and intramuscular preadipocytes were obtained from subcutaneous fat and muscle tissues of 8−day−old Cherry Valley ducks by trypsin digestion and differential adhesion. Isobutylmethylxanthine (IBMX, 0.5 mM), insulin (1 mg/mL), rosiglitazone (RSG) and Dex (Sigma 1 mM, Saint Louis, MO, USA) were used to induce preadipocytes. The subcutaneous fat cells and intramuscular fat cells before induction were recorded as SCP−0 and IMP−0, respectively. Subcutaneous adipocytes and intramuscular adipocytes were induced with an induction system for 4 days, recorded as SCP−4 and IMP−4. All culture systems were performed at 37 °C in 95% humidity with 5% CO_2_ (Thermo Fisher Scientific, Waltham, MA, USA).

### 2.2. RNA Sequencing

After the extraction of total RNA from adipocytes (SCP−0, SCP−4, IMP−0 and IMP−4) with TRIzol, rRNA was removed to preserve mRNA and non−coding RNA (ncRNA). Enriched mRNA and ncRNA are fragmented into short fragments using a fragmentation buffer and reverse transcribed into cDNA with random primers. The second strand of cDNA is synthesized using DNA polymerase I, RNase H, dNTPs (substituting dUTP for dTTP), and buffer. Subsequently, cDNA fragments were purified using the QiaQuick PCR Purification Kit (Qiagen, Venlo, The Netherlands), underwent end repair, Poly(A) tailing, and were ligated to Illumina sequencing adapters. Second-strand cDNA was then digested with UNG (uracil−N−glycosidase). Digestion products underwent fragment selection via agarose gel electrophoresis, PCR amplification, and sequencing on an Illumina HiSeqTM 4000 (or other platform) by Gene Denovo Biotechnology Co., Ltd. (Guangzhou, China).

### 2.3. circRNA Identification and Differential Analysis

First, we used fastp [18] to QC the raw reads and filtered the low−quality data to obtain clean reads. Then we took the HQ clean reads of each sample and used BOWTIE2 (Version 2.2.9) [19] to perform ribosome comparison, and also used HISAT2 (Version 2.2.0) [20] software to compare with the reference genome. The obtained comparison results were submitted to CIRIquant [21] for cyclic RNA identification. For circRNA identification, we filtered the results to obtain highly plausible circRNAs for subsequent analysis. edgeR (Version 3.21) [22] software was used for differential expression analysis of circRNAs. CircRNAs with *p*-value ≤ 0.05 and |log_2_(FC)| ≥ 1 were considered to be significantly different circRNAs. Using intersected software38, the protein-coding transcript with the largest circRNA overlapping region in the genome, i.e., the host gene of circRNA, was obtained based on the number of exons between the circularization site and the shear site in the transcript region.

### 2.4. miRNA Identification

All clean tags were compared to small RNAs in the GeneBank database to identify and remove rRNAs, scRNAs, snoRNAs, snRNAs, and tRNAs. The miRNA sequences were compared with those in the miRBase database to obtain the number of already existing miRNAs and the base distribution. Then, all clean tags were searched in the miRBase database to identify known miRNAs. Known miRNAs were identified by comparing them with miRNAs from other species. The unannotated tag sequences that can be compared to the upper genome were selected using the software MIREAP (Version 0.2), which predicts the specific secondary structure of miRNA to find out the new candidate miRNAs. The criteria of *p*-value ≤ 0.05 and |log_2_(FC)| ≥ 1 were used to screen for significant DE miRNAs.

### 2.5. Target Gene Prediction of DE miRNAs

Prediction of miRNA target genes was performed using RNAhybrid (Version 2.1.2) + svm_light (Version 6.01), Miranda (Version 3.3a), and TargetScan (Version 7.0), and then the intersection of the target gene prediction obtained by the three methods was taken as the result of miRNA target gene prediction. The intersection of the target gene prediction results obtained by the three methods was then taken as the miRNA target gene prediction result.

### 2.6. GO and KEGG Functional Enrichment Analysis

We performed GO (Gene Ontology, http://www.geneontology.org, accessed on 23 July 2025) and KEGG (Kyoto Encyclopedia of Genes and Genomes, http://www.genome.jp/kegg, accessed on 23 July 2025) enrichment analyses on host and indirect target genes of circRNAs. The results with *a P*_value_ ≤ 0.05 were considered to be statistically significant.

### 2.7. Interaction Network Construction

Use the three software tools mireap (Version 2.0), miRanda (Version 3.3a), and TargetScan (Version 2.7) for target gene prediction, and take the intersection of the results obtained from the three software tools as the final prediction results for miRNA target genes. Next, we screened for negatively correlated co-expressed mRNA-miRNA or circRNA−miRNA pairs with a Spearman rank correlation coefficient (SCC) < −0.7, where both mRNA and circRNA were miRNA target genes, and all RNAs were differentially expressed. Since the expression levels of ceRNAs competing for the same miRNA are positively correlated, the Pearson correlation coefficient (SCC) is calculated, and circRNA−mRNA pairs with a correlation coefficient of 0.95 or higher are selected as potential ceRNA pairs. The hypergeometric cumulative distribution function test was used to determine whether the common miRNA sponge between two genes was significant, and circRNA−miRNA−mRNA pairs with *p*-value ≤ 0.05 were selected as the final ceRNA pairs. Finally, the ceRNA network was visualized using Cytoscape software (Version 3.6.0).

### 2.8. Validation of Quantitative Real−Time PCR (qRT−PCR)

qRT−PCR was performed to validate the expression levels of DEcircRNAs and DEmiRNAs; 6 circRNAs and 6 miRNAs were randomly selected from significantly different expression genes in IMP−0, IMP−4, SCP−0 and SCP−4. The primers were designed by Oligo7.0 software and synthesized by Tsingke Biotech Co., Ltd. (Beijing, China) (Table 1). qPCR was performed using the SYBR qPCR Master Mix kit (Vazyme, Shanghai, China) on the LightCycler^®^ 96 system (Roche, Basel, Switzerland). The reaction system consisted of 10 μL 2× FastStart Universal SYBR Green Master, 0.4 μL (10 μM) each of forward and reverse primers, 2 μL cDNA, and 7.2 μL ddH_2_O, resulting in a total reaction volume of 20 μL. miRNA cDNA First Strand Synthesis Kit (AG117160) and SYBR Green Pro Taq HS Premix qPCR Kit II (with ROX, AG11719) (Accurate Biotechnology Co., Ltd., Changsha, China) were used to perform miRNA reverse transcription and quantitative PCR according to their respective protocols. GAPDH was used as the endogenous control of circRNA, and U6 was used as the internal reference for miRNA.

### 2.9. Statistical Analyses

Raw data were imported into Microsoft Office Excel 2016 software. The relative gene expression was calculated by the 2^−ΔΔCt^ method. All experiments were performed in three biological replicates. Differences between groups were analyzed using an independent samples *t*−test in SPSS 22.0 software, with *p* ≤ 0.05 considered statistically significant. Bar graphs illustrating the results were generated using GraphPad Prism 10 software.

## 3. Results

### 3.1. In Vitro Cell Culture and Results of Oil Red O Staining

**Figure 1:**
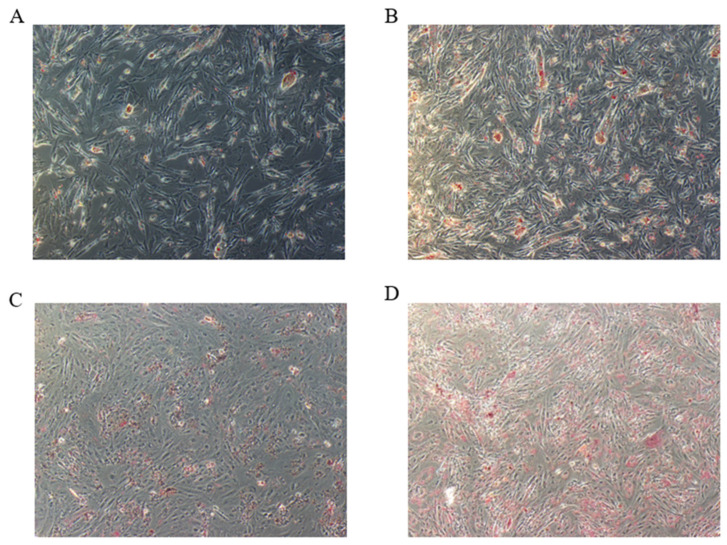
Oil red O staining of intramuscular adipocytes and subcutaneous adipocytes at different times after differentiation. (**A**) Before IMP induction (IMP−0). (**B**) After IMP induction (IMP−4). (**C**) Before SCP induction (SCP−0). (**D**) After SCP induction (SCP−4).

Under in vitro cell culture conditions, phenotypic changes were observed in precursor adipocytes both before and after differentiation induction. Oil Red O staining revealed sporadic lipid droplets emerging within cells starting from day 4 of differentiation induction, with their number gradually increasing over time. Pre−induced precursor intramuscular adipocytes (Figure 1A) and subcutaneous adipocytes (Figure 1B) showed no obvious lipid droplet aggregation; Following differentiation induction, cells progressively accumulated lipid droplets, adopting a more rounded morphology. Initially dispersed small droplets gradually coalesced into larger droplets, exhibiting characteristic red-positive staining. Notably, visible to the naked eye, after day 4 of induction, subcutaneous adipocytes (Figure 1D) displayed obviously more visible red lipid droplets than intramuscular adipocytes (Figure 1C).

### 3.2. Global Responses of circRNAs to Fat Deposition

**Figure 2:**
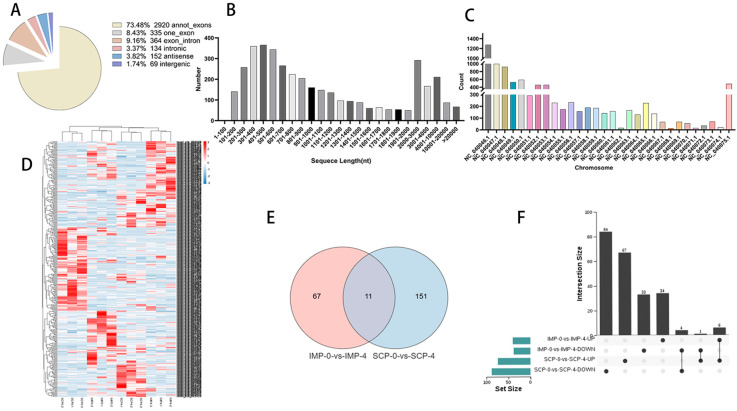
Identification of differentially expressed circRNAs (DEcircRNAs). (**A**) Distribution map of circular RNA types. (**B**) Length distribution map of circular RNAs. (**C**) Chromosomal distribution map of circular RNAs. (**D**) Heatmap of all DE circcRNAs expression in each sample. (**E**) Venn diagrams of circRNAs expressed in IMP−0−vs−IMP−4 and SCP−0−vs−SCP−4 groups. (**F**) Statistical plot displaying up−regulated and down−regulated DE circRNAs.

In order to improve the reliability of circRNA prediction, TopHat and find_circ were used to detect candidate circRNA. The intersection of these 2 tools revealed 3974 circRNAs in IMP−0, IMP−4, SCP−0, and SCP−4, of which 2920, 152, 364, 69, 134, and 335 belonged to annot_exon type, one_exon type, exon_intron type, intron type, antisense type, and intergenic type, respectively (Figure 2A). The sequence length distribution of circRNAs, as shown in Figure 2B, showed that most of the sequences were shorter than 10,000 bp in length. Moreover, the longer the sequences were, the fewer the number of circRNAs were, and the longest circRNA sequence reached 84 kb. We found the most circRNAs on NC_040046.1, followed by NC_040047.1 and NC_040048.1 [Figure 2C]. The heatmap showed the expression profiles of DEcircRNAs in each sample (Figure 2D). After differential expression analysis (Tables S1 and S2), 78 (40 up−regulated, 38 down−regulated) and 162 (74 up−regulated, 88 down−regulated) DEcircRNAs were found in IMP−0−vs−IMP−4 and SCP−0−vs−SCP−4, respectively (Figure 2E). The specific expression of DEcircRNAs was analyzed, and as shown in Figure 2F, 67 DEcircRNAs were specifically expressed in the IMP−0−vs−IMP−4 group, 151 DEcircRNAs were specifically expressed in the SCP−0−vs−SCP−4 group, and 11 DEcircRNAs were expressed in both comparison groups. The top 10 most significantly differentially expressed DEcircRNAs are shown in Table 2 and Table 3.

### 3.3. Functional Annotation of circRNA Host Gene

**Figure 3:**
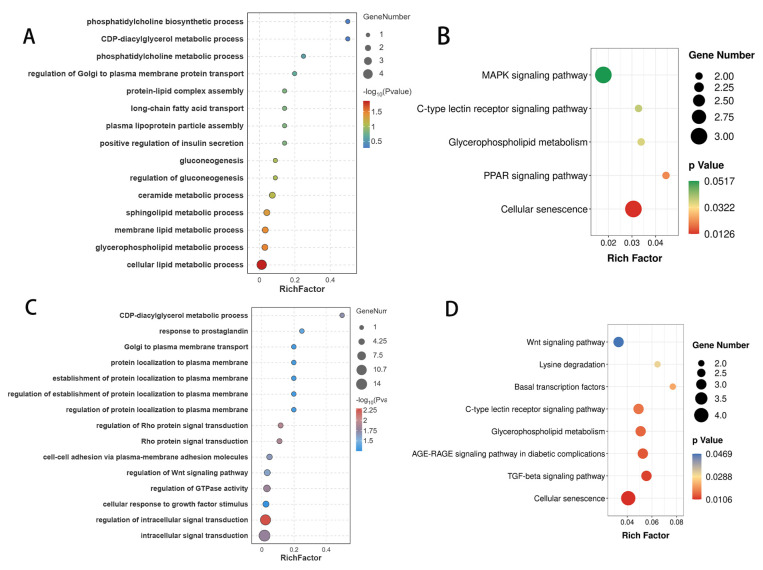
Functional analysis of identified DEcircRNAs. GO Terms and KEGG Pathway. (**A**,**B**) Gene Ontology and Kyoto Encyclopedia of Genes and Genomes in IMP−0−vs−IMP−4. (**C**,**D**) SCP−0−vs−SCP−4.

CircRNAs can affect the transcription of host genes. Therefore, functional enrichment analysis of host genes for DEcircRNAs was performed. In the IMP−0−vs−IMP-4 group, the GO enrichment annotation results showed that the host genes were significantly enriched in long-chain fatty acid transport, protein−lipid complex assembly, cellular lipid metabolic process and other GO terms that are closely related to lipid biological functions (*p*-value ≤ 0.05) (Figure 3A). Moreover, they were also significantly enriched in KEGG pathways for lipid deposition and metabolism, such as the PPAR signaling pathway, Glycerophospholipid metabolism, and MAPK signaling pathway (Figure 3B). Analysis of host genes in the SCP−0−vs−SCP−4 group revealed that GO term and KEGG signaling pathways were significantly involved in the CDP-diacylglycerol metabolic process, the Regulation of Wnt signaling pathway, the Glycerophospholipid metabolism, Wnt signaling pathway, TGF-beta signaling pathway, and other pathways related to lipid synthesis and metabolism (Figure 3C,D).

### 3.4. Global Responses of miRNAs to Fat Deposition

**Figure 4:**
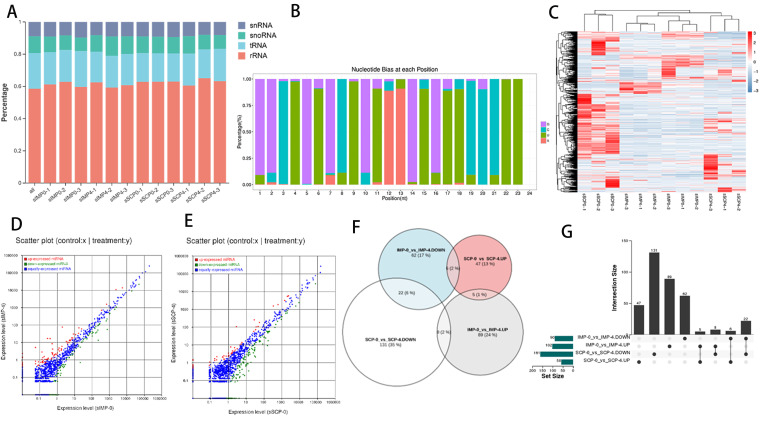
Identification of differentially expressed miRNAs. (**A**) The tag type of miRNAs. (**B**) First base preference of different tag lengths. (**C**) Clustered expression heatmap of all miRNAs. Volcano plot for the identified DELs in IMP−0−vs−IMP−4 (**D**), SCP−0−vs−SCP−4 (**E**), based on the criteria of *p*-value *≤* 0.01 and |log_2_(FC)| ≥ 1. (**F**) Scatter plots of all miRNAs. Red represents SCP−0−vs−SCP−4 up-expressed miRNAs. White represents SSCP−0−vs−SCP−4 down−expressed miRNAs. Blue represents IMP−0−vs−IMP−4 down-expressed miRNAs. Gray represents IMP−0−vs−IMP−4 up−expressed miRNAs. (**G**) Venn diagram showing the up-regulated and down-regulated DE miRNAs.

A total of 1943 miRNAs (1167 known miRNAs and 769 novel miRNAs) were obtained after eliminating other non-coding RNAs such as rRNAs, scRNAs and tRNAs (Figure 4A). The base bias analysis of the obtained preexisting miRNAs showed that the first base of the preexisting miRNA tagged sequences of different lengths was guanine, and they were predominantly expressed at the intermediate (0.01 < percentage < 1) level (Figure 4B). The clustered expression heatmap of the miRNAs showed the differential expression distributions and clustered differentiation (Figure 4C). After differential expression analysis, 192 (102 up-regulated and 90 down−regulated) and 219 (58 up−regulated and 161 down-regulated) DEmiRNAs were found in IMP−0−vs−IMP−4 (Figure 4D and Table S3) and SCP−0−vs−SCP−4 (Figure 4E and Table S4), respectively. After performing intersection and union statistics, 151 DEmiRNAs specifically expressed in the IMP−0−vs−IMP−4 group were present, whereas 178 DEmiRNAs were specifically expressed in the SCP−0−vs−SCP−4 group and 41 DEmiRNAs were found in the intersecting part of the two comparative groups (Figure 4F,G). The top 10 most significantly differentially expressed DEmiRNAs are shown in Table 4.

### 3.5. Functional Annotation of miRNA Target Genes

**Figure 5:**
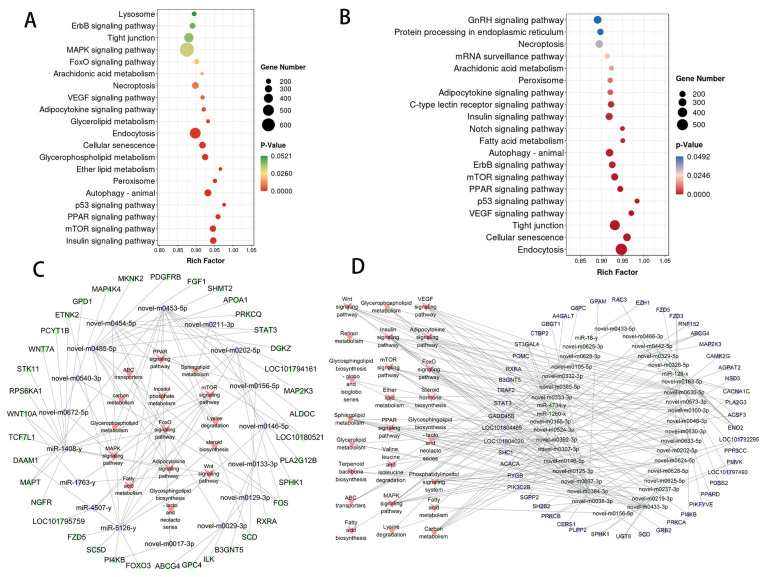
Functional analysis of identified DEmiRNAs. The top 15 KEGG enrichment pathways analysis of DE miRNAs in IMP−0−vs−IMP−4 (**A**) and SCP−0−vs−SCP−4 (**B**). miRNA−mRNA co-expression network of (**C**) IMP−0−vs−IMP−4 (**D**) SCP−0−vs−SCP−4.

To better understand the miRNAs that may contribute to duck adipogenesis, we performed target prediction and functional enrichment analysis of DE-miRNAs. In the IMP−0−vs−IMP−4 group, for 219 DE-miRNAs, a total of 10,838 target genes were identified, of which 9230 target genes were differentially expressed and significantly enriched in terms related to adipogenesis, including Ether lipid metabolism, Glycerophospholipid metabolism, Glycerolipid metabolism and other processes (Figure 5A and Table S5). And 9308 differentially expressed target genes in the SCP−0−vs−SCP−4 group, which were significantly associated with Fatty acid metabolism, Arachidonic acid metabolism, and other lipid metabolism processes (Figure 5B and Table S6).

### 3.6. Integrative Analysis of miRNA and mRNA Expression Profiles

In the present study, we focused on miRNAs that may bind to transcription factors involved in fat deposition. based on the criteria of Energy ≤ −35 kcal/mol, *p*-value ≤ 0.05, score ≥ 1.5, the study targeting the IMP−0−vs−IMP−4 group showed that 181 regulatory pairs (involving 17 DE-miRNAs and 38 differential transcription factors) were identified. Among the lipid metabolism-related pathways, pathways such as steroid biosynthesis and Inositol phosphate metabolism showed significant enrichment (Figure 5C and Table S7). In the SCP−0−vs−SCP−4 group, we identified a total of 313 regulatory pairs involving 39 DE-miRNAs and 50 differentially expressed transcription factors. The lipid-related signaling pathways that were significantly enriched were the Insulin signaling pathway, Glycerolipid metabolism, Steroid hormone biosynthesis, and Retinol metabolism and 24 others (Figure 5D and Table S8).

### 3.7. Construction of the DE circRNA–miRNA–mRNA ceRNA Regulatory Network

**Figure 6:**
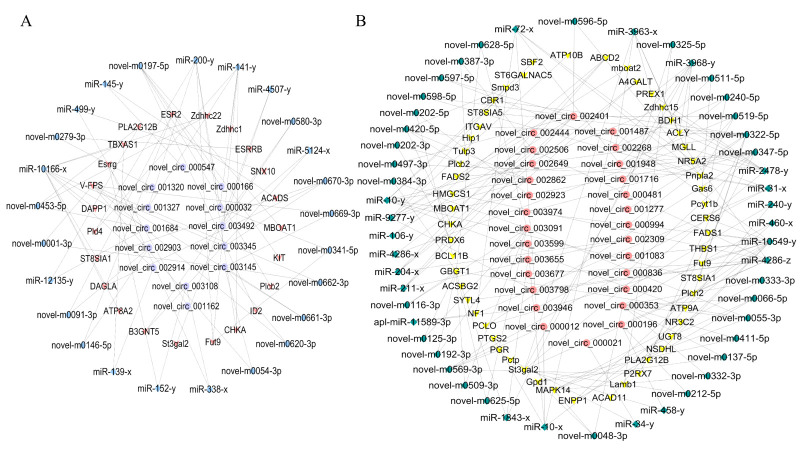
The ceRNA co-regulation network. The ceRNA co-regulation network between the IMP−0−vs−IMP−4 group (**A**) and the SCP−0−vs−SCP−4 group (**B**). The circles, V−shapes and diamonds represent DEcircRNAs, DEmRNAs and DEmiRNAs, respectively.

In the IMP−0−vs−IMP−4 group, 1351 differentially expressed circRNA–miRNA–mRNA pairs were initially identified, comprising 58 differentially expressed circRNAs, 859 differentially expressed mRNAs, and 116 miRNAs. Then, a fat-related ceRNA regulatory network was constructed, including 29 circRNA-miRNA pairs and 40 miRNA-mRNA pairs, involving 23 mRNAs, 13 circRNAs, and 25 miRNAs ((Figure 6A, Table S9). For example, novel_circ_001327 and miR-141-y share the target gene *CHKA*. Similarly, in the SCP−0−vs−SCP−4 group (Figure 6B), 72 circRNA-miRNA pairs and 107 miRNA-mRNA pairs formed 118 fat-related DE circRNA–miRNA–mRNA interactions, involving 50 mRNAs, 28 circRNAs, and 54 miRNAs (Table S9), such as novel_circ_000353/novel-m0048-3p/*MAPK14*.

### 3.8. qRT-PCR Validation of DEcircRNAs and DEmiRNAs

**Figure 7:**
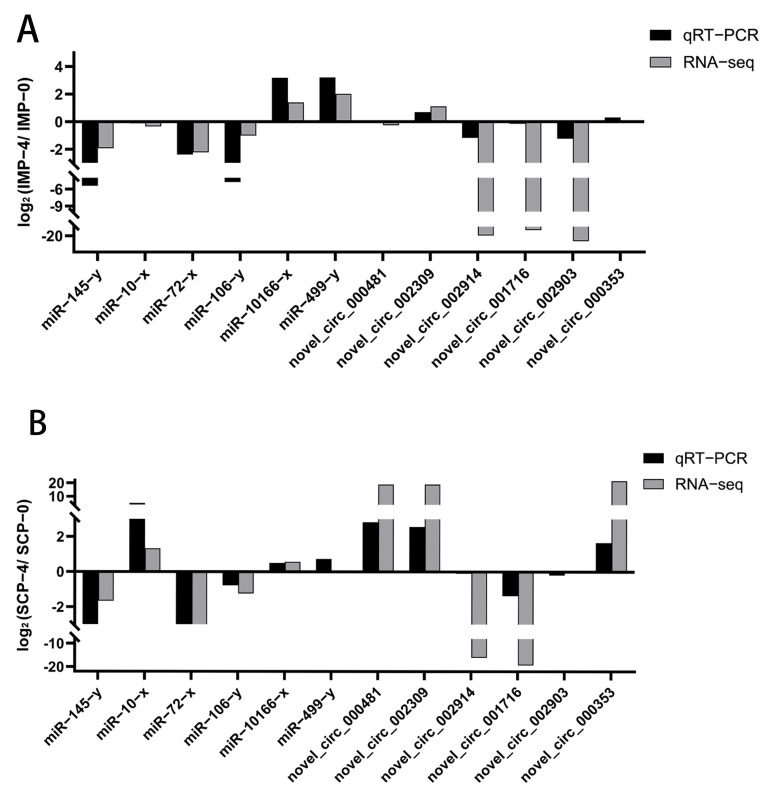
Expressions of 6 miRNAs and 6 circRNAs were validated by qRT−PCR or RNA−seq. (**A**) In IMP. (**B**) In SCP.

Four circRNAs were randomly selected (novel_circ_000481, novel_circ_002309, novel_circ_002914, novel_circ_001716, novel_circ_002903, novel_circ_000353), and six miRNAs (miR−145−y, miR−10−x, miR−72−x, miR−106−y, miR−10166−x, miR−499−y) (Figure S1). The consistency of their expression trends with sequencing results indicates that the gene expression profile data obtained in this study are reliable (Figure 7).

## 4. Discussion

In the livestock product production process, duck meat has many advantages over the meat of mammals such as pigs and cattle, including a short feeding period and a high feed conversion rate [23]. Fat content is an important economic trait of meat ducks, which has a huge impact on the breeding and production of meat ducks, so it has attracted the attention of scientific researchers and producers. The expression patterns, proliferation and differentiation potential, and influence of various regulatory factors in adipocytes from different sources are quite different [4,24,25]. In the abdominal fat and subcutaneous adipose tissue of Peking ducks, differences in the expression of genes related to meat quality traits were found among different parts of adipose tissue, as well as genes related to fat metabolism, such as *STK17A*, *PLIN1*, *PLIN2*, *DGAT2* [26].

There is a high correlation between subcutaneous fat and intramuscular fat content, and in pigs, for every 1% decrease in subcutaneous fat thickness, intramuscular fat content decreases by 0.07% [27], so it is difficult to reduce body fat deposition and increase intramuscular fat content through traditional breeding methods. Compared with intramuscular precursor adipocytes, subcutaneous precursor adipocytes proliferate slowly and are large in size, but have a high degree of differentiation [28,29]. The adipogenic differentiation capacity of intramuscular adipocytes is significantly lower than that of subcutaneous adipocytes, as evidenced by lower expression levels of genes associated with adipogenesis and higher expression levels of genes associated with lipolysis in intramuscular adipocytes. It indicates that the deposition of intramuscular and subcutaneous fat in animals is site−specific [25]. There are physiological particularities in the differentiation and lipid metabolism capacity of subcutaneous fat, and this difference provides a biological basis for the search for the specific differentiation and regulation mechanism of fat metabolism in subcutaneous adipocytes.

Circular RNAs (circRNAs) and microRNAs (miRNAs) play pivotal roles in regulating fat deposition in livestock and poultry. Regarding circRNAs, they demonstrate core regulatory functions in lipogenesis across different species. For instance, in ducks, circ−PLXNA1 is primarily expressed in adipose tissue and exerts significant influence on lipogenesis by downregulating *DLK1* while simultaneously upregulating *C/EBPα* and *FAS* expression levels [30]. circRNAs can also regulate lipogenesis through ceRNA networks. For instance, in chickens, circDOCK7 acts as a “molecular sponge” by binding gga−miR−301b−3p, thereby releasing its inhibition on *ACSL1* and synergistically promoting preadipocyte proliferation and differentiation [31]. Beyond circRNAs, miRNAs have been identified as key regulators of fat deposition, particularly in tissue-specific differential fat accumulation. Studies indicate that miR−27b reduces intramuscular fat deposition by targeting *PPARγ*, thereby participating in the regulation of subcutaneous versus intramuscular fat distribution [32]. Such tissue-specific regulation is particularly pronounced in pig studies: miR−199a−5p exhibits distinct dynamic expression patterns in the longissimus dorsi muscle and subcutaneous fat, and its overexpression specifically inhibits lipid synthesis in intramuscular adipocytes [33]; conversely, miR−32−5p exerts a stronger inhibitory effect on intramuscular fat deposition than on subcutaneous fat, potentially due to its higher expression levels in intramuscular fat [34]. In summary, although the roles of circRNAs and miRNAs in regulating fat deposition, particularly in mediating tissue-specific differentiation, have been confirmed in multiple livestock species, the genome-wide expression profiles, key ceRNA networks, and functional mechanisms of these molecules in the differential deposition of intramuscular and subcutaneous fat in meat ducks remain largely unexplored [35].

In the IMP−0−vs−IMP−4 group, circRNA host genes were significantly enriched in long-chain fatty acid transport and protein-lipid complex assembly. Upregulation of long-chain fatty acid transport-related genes may promote the uptake of circulating fatty acids by intramuscular adipocytes, providing a molecular basis for their function as a “metabolic buffer pool”. The activation of the PPAR signaling pathway further confirms the central position of this fat reservoir in the regulation of lipid metabolism. In contrast, subcutaneous fat differentiation (SCP−0−vs−SCP−4) showed unique regulatory characteristics, and the enrichment of CDP−diacylglycerol metabolism and glycerol phospholipid metabolism pathways suggested that subcutaneous fat was more inclined to membrane lipid synthesis and cell structure remodeling. The significant involvement of Wnt and TGF−β signaling pathways may maintain homeostasis in subcutaneous adipose tissue by inhibiting excessive fat differentiation, which is compatible with its functional needs as long−term energy storage organs. We speculate that the process of intramuscular adipocyte differentiation may be achieved by activating lipid transport and metabolic pathways (e.g., MAPK signaling) to respond quickly to local energy demands. The differentiation process induced by subcutaneous adipocytes focuses on lipid synthesis and differentiation regulation (such as the Wnt pathway) to maintain tissue structure and long-term energy reserves. These functional differences reflect different molecular strategies for cellular differentiation of the two adipose tissues, which may provide new targets for targeted regulation of adipose differentiation. Future work may optimize the metabolic function of intramuscular fat by targeting IMP−specific circRNAs, such as molecules that regulate long-chain fatty acid transport; Simultaneous regulation of Wnt-related circRNAs in SCP is expected to improve the distribution and volume of subcutaneous fat.

After removing other non−coding RNA interferences, we identified 1943 miRNAs, 769 of which were novel, a discovery that significantly expanded the miRNA annotation library in the field of lipidobiology. All known miRNAs showed 5′−terminal guanine (G) preference, which was consistent with the recognition specificity of Drosha enzymes for G bases during typical miRNA processing, suggesting a conserved miRNA biosynthesis mechanism in adipose tissue. In the IMP−0−vs−IMP−4 group, there was a relatively balanced miRNA regulation, suggesting a bidirectional regulatory mechanism. In the SCP−0−vs−SCP−4 group, there is a significant downregulation tendency, possibly because many miRNAs may be “active” before differentiation, maintaining the undifferentiated state of preadipocytes by inhibiting these key differentiation genes, and when differentiation is induced, these inhibitory miRNAs need to be downregulated to release the inhibition of target genes and allow the normal expression of differentiation−related genes. The metabolic characteristics of the intramuscular fat-induced differentiation process are mainly related to the dynamic balance of membrane lipids, which may be to maintain the fluidity of intramuscular fat cell membranes to adapt to the mechanical stress of muscle contraction, thereby improving the meat texture of meat ducks. Subcutaneous fat differentiation mainly involves fatty acid storage and mobilization, which may provide a new direction for reducing the deposition of subcutaneous fat in meat ducks.

This study systematically constructed a competitive endogenous RNA (ceRNA) network to reveal the dynamic changes in the circRNA−miRNA−mRNA regulatory axis during the differentiation of intramuscular fat (IMP) and subcutaneous fat (SCP) in ducks. In the ceRNA networks of the IMP−0−vs−IMP−4 and SCP−0−vs−SCP−4 groups, the involved circRNAs and miRNAs exhibit tissue specificity, while the target genes are both tissue−specific and non-specific. There are nine overlapping genes: *FUT9*, *CHKA*, *PLCB2*, *MBOAT1*, *PLA2G12B*, *STBSIA1*, and *ST3GAL*. The ceRNA network identified in IMP−0−vs−IMP−4 exhibits a relatively streamlined regulatory architecture. A single circRNA contains multiple miRNA response elements (MREs), such as novel_circ_001327, which binds to miR−141−y and miR−200−y, thereby alleviating the inhibition of *Pld4*, *Fut9*, and *CHKA*, and is significantly enriched in the pathways of glycosphingolipid biosynthesis, glycerophospholipid metabolism, and ether lipid metabolism. Additionally, miR−141−y serves as one of the core hubs in the network, connecting two circRNAs and four adipogenesis-related mRNAs (*Pld4*, *Fut9*, *CHKA*, and *Zdhhc1*), among which *Zdhhc1* exhibits tissue−specific changes before and after intramuscular fat induction. Further validation of the regulatory axis involving novel_circ_001327-miR−141−y−Zdhhc1 is warranted. In the SCP−0−vs−SCP−4 ceRNA network, higher complexity is observed, forming a densely interconnected network. Novel_circ_002268 is one of the core regulatory factors, with the highest connectivity in the entire network. By binding to four miRNAs (miR−2478−y, miR−3963−x, miR−3968−y, novel−m0240−5p), it influences eight key genes (*MGLL*, *ACLY*, *BDH1*, *MGLL*, *Pcyt1b*, *PREX1*, *St3gal2* and *Zdhhc15*), primarily by regulating lipid metabolism and cellular signaling pathways, thereby influencing the development and function of subcutaneous fat. Based on this, we speculate that novel_circ_002268 may upregulate *ACLY* by inhibiting miR−2478−y or miR−3963−x, potentially inhibiting lipolysis and promoting subcutaneous fat accumulation, but this requires further validation.

## 5. Conclusions

Herein, a total of 78 circRNAs and 151 miRNAs were identified before and after intramuscular adipocyte induction, while 162 circRNAs and 178 miRNAs were identified before and after subcutaneous adipocyte induction, thereby enriching the RNA library of meat ducks. Additionally, a DEcircRNA-miRNA-mRNA ceRNA network was constructed. Regulatory networks such as novel_circ_001327/miR−141−y/*Zdhhc1* were identified in IMP−0−vs−IMP−4, while networks including novel_circ_002268/miR−2478−y (or miR−3963−x)/*ACLY* regulatory network in SCP−0−vs−SCP−4. Preliminary inferences suggest these networks may, respectively, regulate intramuscular fat and subcutaneous fat deposition and metabolism, though further experimental validation is required. It is anticipated that these candidate circRNAs, miRNAs, and ceRNAs will play a significant role in elucidating the specific deposition of intramuscular and subcutaneous fat in meat ducks and in enhancing meat quality. Currently, this study relies primarily on bioinformatics-based predictions rather than robust functional experimental evidence. Further validation of key ceRNA axes through cellular and animal experiments is required.

## Figures and Tables

**Table 1:** Primer sequences of qRT-PCR.

Gene	Primer Sequence (5′–3′)
U6	F: GGAACGATACAGAGAAGATTAGC R: TGGAACGCTTCACGAATTTGCG
GAPDH	F: AGATGCTGGTGCTGAATACG R: CGGAGATGATGACACGCTTA
novel_circ_000481	F: ATGTGCTTGCACCTATGGCT R: AGCTACCTCCGTTCATGCAC
novel_circ_002309	F: CGCTTTGTCCGAGGAGACAT R: TTGTATGCCACTGTCAACGC
novel_circ_000353	F: CAGCCTCAGTCCGAATGGTT R: AGCCAGGAATGTCAGATGCC
novel_circ_002903	F: CGCTTTGTCCGAGGAGACAT R: TTGTATGCCACTGTCAACGC
novel_circ_002914	F: TGCCAGCACTAACAATTTGCC R: ATACAGCCATCCCCATTATCCG
novel_circ_001716	F: GGTCTGCTTTTCCATTGGGC R: TTCAAGCCAGCTAACACCGT
miR-145-y	GCGCGATTCCTGGAAATACT
miR-10166-x	GCGCGTGCAGCTGATGA
miR-72-x	AGGCAAGATGTTGGCATAGCTG
miR-106-y	CGCGACTGCAGTATAAGCACT
miR-10-x	TACCCTGTAGATCCGAATTTGT
miR-499-y	CGCGCGCGAACACACA

**Table 2:** The top 10 circRNAs with the most significant expression level differences between the IMP−0 and IMP−4 groups (absolute values of upregulation and downregulation).

circRNA−ID	log_2_(FC)	*p*-Value	Source_Gene
novel_circ_002903	−21.98876634	1.64 × 10^−6^	ncbi_101798557
novel_circ_001112	−21.06765464	1.84 × 10^−4^	ncbi_101796118
novel_circ_001327	−20.75001229	5.53 × 10^−5^	ncbi_101798557
novel_circ_003145	−20.4450971	4.46 × 10^−4^	ncbi_101797443
novel_circ_000955	−20.09971448	9.18 × 10^−3^	ncbi_101790241
novel_circ_000347	−20.02493364	1.45 × 10^−2^	ncbi_101799066
novel_circ_002914	−19.95022905	5.97 × 10^−3^	ncbi_101799066
novel_circ_000683	20.40296203	2.61 × 10^−3^	ncbi_101792777
novel_circ_003292	20.40454937	4.34 × 10^−3^	ncbi_101802634
novel_circ_002224	20.44693427	4.67 × 10^−4^	ncbi_101789710
novel_circ_003570	20.45954433	1.80 × 10^−3^	ncbi_101792634

**Table 3:** The top 10 circRNAs with the most significant expression level differences between the SCP−0 and SCP−4 groups (absolute values of upregulation and downregulation).

circRNA−ID	log_2_(FC)	*p*-Value	Source_Gene
novel_circ_002525	−20.33703397	2.16 × 10^−5^	ncbi_101790166
novel_circ_002703	−20.32357405	3.65 × 10^−5^	ncbi_106015233
novel_circ_002444	−20.28053857	2.43 × 10^−5^	ncbi_101792223
novel_circ_003697	−20.0386573	4.15 × 10^−4^	ncbi_101790695
novel_circ_001312	−20.00303916	1.75 × 10^−4^	ncbi_101801709
novel_circ_002176	−19.95397623	5.67 × 10^−4^	ncbi_101805096
novel_circ_001016	−19.89744	2.99 × 10^−4^	ncbi_101803584
novel_circ_002829	−19.79543522	3.50 × 10^−3^	ncbi_101795905
novel_circ_003429	20.69082673	2.60 × 10^−4^	ncbi_101800418
novel_circ_000353	21.82125304	4.26 × 10^−14^	ncbi_101798353

**Table 4:** The top 10 miRNAs with the most significant expression level differences between the IMP−0−vs−IMP−4 group and the SCP−0−vs−SCP−4 group (absolute values of upregulation and downregulation).

IMP−miRNA−ID	IMP−log_2_(FC)	IMP-*p*-Value	SCP−miRNA−ID	SCP−log_2_(FC)	SCP-*p*-Value
novel-m0001-5p	−6.912210731	9.15 × 10^−6^	novel-m0355-5p	−9.925366519	5.55 × 10^−16^
novel-m0054-5p	−6.594946589	6.73 × 10^−5^	novel-m0336-5p	−8.617798026	1.62 × 10^−8^
novel-m0620-5p	−6.5360529	1.52 × 10^−4^	novel-m0630-3p	−8.035935434	9.30 × 10^−6^
novel-m0661-5p	6.503235273	2.54 × 10^−14^	novel-m0338-5p	−7.726513368	7.56 × 10^−5^
novel-m0662-5p	6.503235273	2.42 × 10^−14^	miR-240-y	−7.700023496	7.31 × 10^−5^
novel-m0669-5p	6.503235273	2.11 × 10^−14^	novel-m0301-3p	−7.680793175	7.41 × 10^−5^
novel-m0298-5p	7.070961712	5.20 × 10^−8^	novel-m0241-5p	−7.364776683	1.40 × 10^−3^
novel-m0212-5p	7.203266423	8.60 × 10^−8^	novel-m0267-5p	−7.276341569	6.78 × 10^−4^
novel-m0589-5p	7.852935186	5.05 × 10^−11^	novel-m0618-5p	−7.276341569	7.08 × 10^−4^
novel-m0670-5p	7.898833127	4.13 × 10^−10^	miR-10549-y	7.256664742	1.28 × 10^−4^

## Data Availability

All datasets generated or analyzed during this study are available from the corresponding author on reasonable request.

## Supplementary Materials

The following supporting information can be downloaded at: https://www.mdpi.com/article/10.3390/genes16101208/s1, Table S1. Table of differential circRNAs analysis in the IMP−0−vs−IMP−4 group results; Table S2. Table of differential circRNAs analysis in the SCP−0−vs−SCP−4group results; Table S3. Functional annotation of miRNA target genes in IMP−0−vs−IMP−4; Table S4. Functional annotation of miRNA target genes in SCP−0−vs−SCP−4; Table S5. DemiRNAs of IMP-0-vs-IMP-4; Table S6. DemiRNAs of SCP−0−vs−SCP−4; Table S7. Comprehensive Analysis of miRNAs and mRNAs in IMP−0−vs−IMP−4; Table S8. Comprehensive Analysis of miRNAs and mRNAs in SCP−0−vs−SCP−4; Table S9. ceRNA network of IMP−0−vs−IMP−4; Table S10. ceRNA network of SCP−0−vs−SCP−4. Figure S1. The results of the real-time quantitative PCR validation of the expression level. The expression level of miRNAs in the IMP−0−vs−IMP−4 group (**A**) and SCP−0−vs−SCP−4 (**B**) group were validated by RT-qPCR. The expression level of circRNAs in the IMP−0−vs−IMP−4 group (**C**) and SCP−0−vs−SCP−4 (**D**) group were validated by RT-qPCR. ** indicates *p ≤* 0.01, * indicates *p ≤* 0.05. The data represented the mean ± SEM from three biological replicates, and each measurement was repeated 3 times.
