# Supplementary figures and images for: Comprehensive Identification of miRNAs and circRNAs in the Regulation of Intramuscular and Subcutaneous Fat Deposition in Meat Ducks
Source: Genes (Basel). 2025 Oct 14;16(10):1208. doi: 10.3390/genes16101208 (PMC12562460; doi:10.3390/genes16101208)

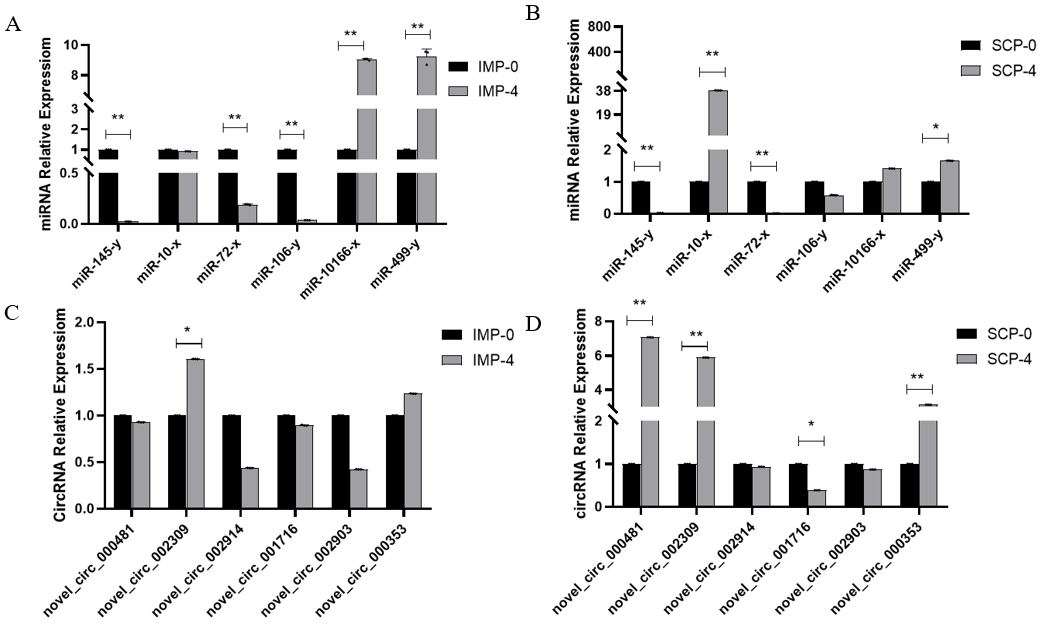

Supplement: Supplementary file 1 [file genes-16-01208-s001.zip › Figure S1 The results of the real-time quantitative PCR validation of the expression level..png]
